# Three-Dimensional Printed Biomimetic Elastomeric Scaffolds: Experimental Study of Surface Roughness and Pore Generation

**DOI:** 10.3390/biomimetics10020095

**Published:** 2025-02-08

**Authors:** Daniele Marazzi, Federica Trovalusci, Paolo Di Nardo, Felicia Carotenuto

**Affiliations:** 1 Department of Clinical Sciences and Translational Medicine, University of Rome Tor Vergata, 00133 Rome, Italy; daniele.marazzi@uniroma2.it; 2 Department of Enterprise Engineering, Mario Lucertini University of Rome Tor Vergata, 00133 Rome, Italy; federica.trovalusci@uniroma2.it; 3 Interdepartmental Center for Regenerative Medicine (CIMER), University of Rome Tor Vergata, 00133 Rome, Italy; dinardo@uniroma2.it

**Keywords:** biomimetic scaffold, elastomeric resin, surface porosity, roughness, printing orientation, additive manufacturing, stereolithography

## Abstract

Tissue engineering is an emerging field within biomedicine, related to developing functional substitutes for damaged tissues or organs. Despite significant advancements, the development of effective engineering tissue constructs remains challenging, particularly when replicating elastic stretchability, which plays a critical role in many tissues. Therefore, the development of tough, elastomeric scaffolds that mimic the complex elasticity of native tissues, such as the myocardium, heart valves, and blood vessels, is of particular interest. This study aims to evaluate a flexible printable material (Formlabs’ Elastic 50A Resin V2) to develop porous 3D scaffolds using additive manufacturing stereolithography (SLA). The elastomeric samples were tested in relation to their swelling behaviour, mechanical properties, and exposure to low temperatures. Additionally, the effects of print orientation, water immersion, and exposure to low temperatures on surface roughness and porosity were investigated to determine the best conditions to enhance scaffold performance in biomedical applications. The results demonstrated that samples printed at 0°, immersed in water, and exposed to low temperature (−80 °C) showed a more uniform microporosity, which could improve the adhesion and growth of cells on the scaffold. This research highlights a practical and economical approach to enhancing elastomeric scaffolds, paving the way for improved outcomes in tissue engineering applications.

## 1. Introduction

Tissue engineering is a growing interdisciplinary field that merges biology, engineering, and medicine to repair and regenerate tissues [1,2]. Central to this discipline is the design and fabrication of three-dimensional (3D) scaffolds, which mimic the extracellular matrix (ECM) of biological tissues and serve as critical structural frameworks essential for cellular proliferation and differentiation. Material selection plays an important part in the design and production of scaffolds for successful regenerative therapy. Since elastic deformability is a fundamental mechanical property of many tissues, significant efforts have been dedicated to developing elastomeric biomaterials that replicate the properties of native tissues. In this field, the development of tough, synthetic biodegradable amorphous elastomers that can exhibit rubberlike elasticity is of particular interest [3,4].

Despite significant improvements, engineering effective tissue constructs based on synthetic elastomers remains challenging, particularly in terms of replicating the intricate physiological environments necessary for optimal cellular behaviour [5,6].

One of the main obstacles to tissue regeneration is the suboptimal microenvironment provided by conventional scaffolds, which often fails to facilitate adequate cell adhesion, proliferation, and differentiation [7]. This inadequacy can lead to unfavourable clinical outcomes, including incomplete tissue regeneration and fibrotic scarring [8]. To improve the interactions between cells and biomaterials, scaffold surface modification is often employed [9]. Such modifications are instrumental in improving cell adhesion and delivering biochemical signals that promote critical cellular activities, thereby increasing the effectiveness of regenerative processes [10]. This is particularly crucial in clinical applications where the precise modulation of cellular behaviour is essential for successful therapeutic outcomes [10,11].

A key consideration in scaffold design is the facilitation of cell proliferation within a supporting microenvironment. Scaffolds can adopt simplified geometries or complex architectures that closely mimic the anatomical structures of native tissues [12]. The design process must meticulously take into account the mechanical properties dictated by the selection of materials, as well as the biological attributes relevant to the target tissue type [13]. These considerations are critical to ensuring that scaffolds can withstand physiological loads while also promoting effective tissue regeneration [14].

In this context, additive manufacturing (AM) has emerged as a transformative technology for biomechanical applications, enabling the production of highly customized scaffold geometries tailored to specific tissue needs [15]. AM techniques offer precise control over microstructural attributes and mechanical properties, significantly improving scaffold performance in terms of supporting tissue regeneration [16]. Among the various AM modalities, stereolithography (SLA) is particularly suitable for fabricating scaffolds with complex geometric configurations, essential for replicating the intricate architectures of biological tissues [17].

However, in addition to mechanical precision, biological characteristics such as surface porosity profoundly influence the performance of the scaffold [18]. Porous scaffolds are essential for mimicking the ECM, facilitating cell adhesion, and providing an optimal environment favourable to growth and differentiation [19,20]. Adequate porosity promotes essential processes such as cell infiltration, nutrient diffusion, and the removal of metabolic wastes, vital mechanisms for sustaining cell viability within the scaffold structure [21,22].

The design of porous architecture requires meticulous attention to the size and geometry of the pores; pores should be large enough to allow cell infiltration but small enough to maintain structural integrity under physiological conditions [23]. In addition, pore geometry can have a significant impact on cell growth directionality and angiogenesis, critical factors that influence the integration of regenerated tissues with host systems. Porosity customization can be achieved through advanced computational design tools or specific post-fabrication treatments [24,25].

Material selection is equally crucial in scaffolding design. The materials chosen must have mechanical and biological properties compatible with the target tissue type [26]. Recent advances have led to the development of a wide range of polymeric materials suitable for the fabrication of scaffolding, classified into synthetic polymers and natural biomaterials, each with distinct profiles of biocompatibility and biodegradability [27]. Biodegradable polymers such as polylactide acid (PLA) and polyglycolide acid (PGA) are often used for soft tissue applications due to their ability to degrade at rates that favour the formation of new tissues without compromising the integrity of the scaffold [22,28].

However, their inherent stiffness limits their applicability to soft and elastic tissues such as the myocardium, heart valves, and blood vessels [29]. As a result, elastomeric materials have been designed for their ability to withstand repetitive load cycles while maintaining stable mechanical properties over time [30,31]. However, the us of elastic printable materials for medical applications has not been fully explored.

Therefore, this research seeks to assess an elastic printable and commercially available material for the creation of porous 3D scaffolds, utilizing SLA technology, with the goal of mimicking the biomechanical properties found in soft tissues. The elastomeric samples were evaluated for swelling behaviour, mechanical properties, and exposure to low temperatures.

Subsequently, to create a porous surface architecture, an analysis was conducted to evaluate how printing orientation, water immersion, and exposure to low temperatures affect the surface of the elastic material.

Scaffolds were designed with simplified geometries, printed using three different orientations relative to the print bed (0°, 45°, and 90°), to analyze how printing orientation affects porosity and pore distribution.

Then, the printed scaffolds were immersed in water to facilitate hydration. Finally, the scaffolds were exposed to two sequential low temperatures at −20 °C and −80 °C, enabling the volumetric expansion of the absorbed water and promoting pore formation. These temperatures were specifically selected based on the resin’s glass transition temperature (Tg) of −34.5 °C [32]. Operating near and below the Tg facilitates structural changes in the material, as the reduced molecular mobility at these temperatures enhances the expansion effect of the absorbed water within the material scaffold. The results of these analyses could be useful for developing a simple and cost-effective method to create porous elastomeric scaffolds that potentially promote cell adhesion and tissue regeneration [33,34].

## 2. Materials and Methods

Elastic 50A Resin V2 (Formlabs Inc., Somerville, MA, USA), an elastomeric resin specifically designed for the fabrication of flexible 3D mockups using AM technology, was used to produce the scaffolds. The composition of the resin is shown in Table 1, with a density of 1.01 g/cm^3^, a dynamic viscosity of 1400 mPa·s, a measurement point of 35 °C, and a temperature Tg of −34.5 °C, and data are taken from the material’s datasheet [32].

To test the swelling profile, the elastomeric samples were made using a reverse engineering process, which involved the production of a mould using AM SLA technology. Subsequently, the resin was poured into the mould and light-cured in a UV oven. This methodology was adopted to ensure more consistent results, eliminating possible variations caused by the printing process. Finally, the specimens were placed in Petri dishes and subjected to immersion in water or in saline solution (NaCl 0.9%) at a physiological temperature (37 °C). For each specimen, the weight change was calculated. The experiment was conducted on six specimens for each duration and type of immersion liquid.

Swelling (S) was calculated according to the following Equation (1), where *W_dry_* is the mass of dry sample before the immersion and *W_wet_* is the mass of the sample after immersion in water for different durations.

(1) $$S=\left(\frac{W_{wet}-W_{dry}}{W_{dry}}\right)\times 100$$

A tensile test was carried out to analyze the changes in mechanical properties as a function of immersion times, in accordance with ASTM D412, specific to elastomeric materials. The experiment was conducted using an MTS Insight 5 traction machine, equipped with a 100 N load cell (MTS Systems Corporation, Eden Prairie, MN, USA). The choice of a low-load cell was aimed at ensuring high sensitivity, allowing for the precise detection of small changes in mechanical properties induced by the treatment.

The specimen, with a “dog bone” geometry, was fabricated using SLA additive manufacturing (AM) technology and oriented parallel to the print bed. This configuration was selected to allow the accurate analysis of the variations in the elastic modulus, representative of the behaviour of the material in relation to the specific orientation and the printing parameters adopted.

The main parameters used for the tensile test are shown in Table 2.

A Zortrax Inkspire SLA 3D printer (Zortrax, Warsaw, Poland) was utilized to create 3D-printed samples featuring simplified geometries (measuring 10.00 × 10.00 × 2.00 mm). These samples were printed in three different orientations relative to the print bed: 0°, 45°, and 90° (six samples for orientation).

The examination of these samples enabled us to establish a connection between the printing angle and surface characteristics, with the aim of developing a porous surface structure. In addition, an analysis was conducted to examine how aqueous solution immersion and exposure to low temperatures influence surface roughness and the generation of porosity. The target pore size ranged from 10 to 50 μm, facilitating the accommodation of myocytes and the formation of microvascular networks within the scaffolds [35].

A Mitaka PF 60 Digital Laser Profilometer and a Roughness Tester (Mitaka Kohki Co., Ltd.,Tokyo, Japan) were used to verify the surface roughness of the fabricated samples. Image acquisition was performed using a Hirox digital 3D microscope (Hirox Co., Ltd., Tokyo, Japan) equipped with a lens with a maximum magnification of 160×.

**Figure 1:**
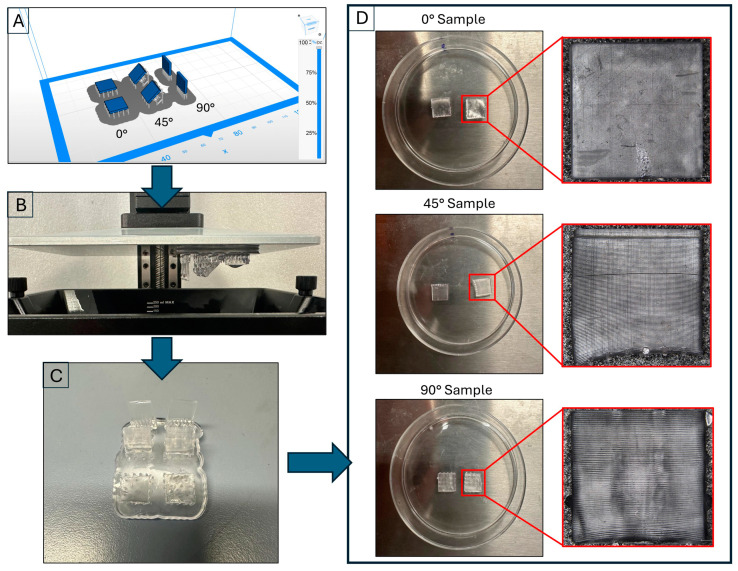
Main steps to produce the specimens: (**A**) slicing phase; (**B**) final stage of production; (**C**) post-processing; (**D**) specimen placed in Petri dish for the immersion phase.

Starting from the CAD model (SolidWorks), an STL file was generated and subsequently processed using slicing software (ZSuite 2.32.00). The print parameters were previously developed by us [36] and are reported in Table 3. Specimens were printed using a single job to ensure production consistency, as shown in Figure 1A. In addition, as specified in Table 3, a variable density of supports was set to achieve an optimal compromise between the sample support and the minimization of surface defects. Overall, 3.77 mL of resin was used for the job, compared to 3.23 mL estimated by the slicing software, with a margin of error of 14.32%. The resulting time to print the samples was 2 h and 23 min.

The specimens underwent post-processing that included ultrasonic cleaning in isopropyl alcohol for five minutes to remove any uncured resin residue. They were then dried using compressed air. Figure 1C shows the samples at the end of this phase. Finally, the samples were placed in 50 mm diameter Petri dishes, as illustrated in Figure 1D.

At each phase of the treatment, the samples underwent surface scans to analyze morphology evolution using a Mitaka noncontact digital roughness tester (Mitaka Kohki Co., Ltd., Tokyo, Japan). The scan length was set to 2.00 mm, matching the sample thickness to capture critical surface changes without introducing measurement distortions. This length was optimal for detecting relevant structural features like pore formation and microcracks, balancing measurement accuracy with the need for representative data. The profiles from six samples for each orientation were examined to evaluate the average linear roughness, with 2D scanning parameters detailed in Table 4.

For a more in-depth analysis of morphology, 3D reconstruction of the surface was performed. These analyses made it possible to trace the evolution of the surface, determining the depth of pores. The acquisition parameters used for the generation of the 3D maps are reported in Table 5.

## 3. Results and Discussion

In this study, an elastic printable material (Elastic 50A Resin V2, Formlabs) was assessed in relation to its usability in the tissue engineering field in order to manufacture scaffolds.

In the first phase, the elastomeric samples were evaluated after immersion in both water and saline solution at 37 °C to mimic the biological environment. The mechanical properties were also studied after water immersion and exposure to low temperatures.

Subsequently, an analysis was carried out on the effect of printing orientation, water immersion, and low-temperature exposure on the surface roughness and porosity generation.

### 3.1. Swelling Analysis

To assess the elastomeric scaffold swelling profile the samples, were created through a reverse engineering process that included the production of a mould using AM SLA technology, as described in the Section 2. Analysis was carried out using both distilled water and a 0.9% NaCl solution (saline solution) to evaluate the behaviour of the material during immersion at 37 °C for different periods of soaking, as shown in Table 6. For each duration, we measured the weight of the samples compared to the reference samples that were kept dry (not immersed) (Table 6).

The results obtained indicate that, after two days of immersion, the elastomeric material absorbs a quantity of liquid equal to 3.36 ± 0.71% in water and 1.59 ± 0.04% in saline solution, while the swelling degree for the samples tends to increase after 3, 5, and 7 days for both types of liquid.

However, it can be observed that swelling is more pronounced in the samples immersed in water, while it appears more contained in those immersed in saline solution. Thus, the swelling efficiency of the elastomeric scaffolds was higher in water than in the physiological solution medium for all immersion times considered.

These outcomes suggest that subsequent experiments should employ distilled water to immerse the 3D-printed samples produced by AM SLA technology with the goal of creating a porous structure in the samples.

In fact, the elastomeric samples absorbed a greater amount of water than the saline solution, which could create a porous morphology in the scaffolds by freezing at low temperatures and then increasing in volume.

### 3.2. Study of Mechanical Properties

The variation in mechanical properties as a function of immersion times in water was also investigated. In light of the preliminary results, it was decided to proceed with the immersion of the specimens exclusively in distilled water in order to evaluate the variation in Young’s modulus resulting from immersion. As shown in Table 7, three different dive durations were considered: two, five and seven days. We also performed a reference test on non-immersed specimens (dry samples), indicated as 0 days.

For each interval, three specimens with a “dog bone” geometry, made using additive manufacturing (AM) SLA technology, were immersed in Petri dishes containing water and then placed in a stove at a constant temperature of 37 °C, corresponding to the physiological temperature.

For the calculation of the percentage change in Young’s modulus (*E*_%_), the Equation (2) was used.

(2) $$E_{\%}=\frac{E_{ref}-E_{i}}{{\;E}_{ref}}\times 100$$

where *E_ref_* represents the value of the reference Young’s modulus, while *E_i_* represents the value of Young’s modulus at dive time.

**Figure 2:**
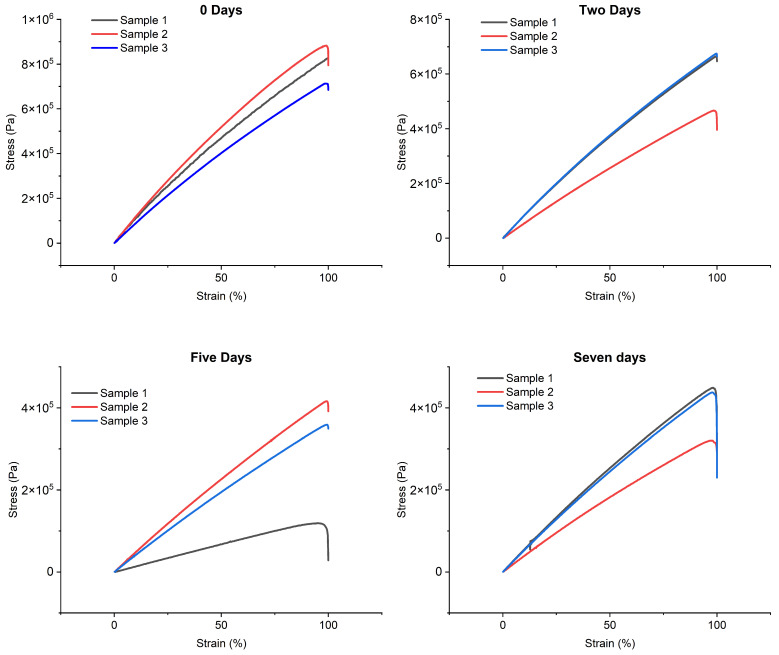
Trends in the stress–strain curves as a function of the dive time.

Figure 2 shows the stress–strain curves obtained from tensile tests using the immersion times and reference values.

Analyzing the results, it can be observed that, after two days of water immersion, Young’s modulus underwent a significant reduction, equal to 27.02%. This decrease continued in the following days, reaching a decrease of 53.43% after five days. After this phase, a stabilization trend was observed, with an overall reduction of 50.31% on the seventh day, suggesting a convergence phase of the Young’s modulus.

Based on these results, a two-day immersion of the samples in water was hypothesized to cause sufficient water absorption while maintaining more contained variation in Young’s modulus. This approach should ensure that the changes induced on the material by water immersion remain within acceptable limits, preserving adequate mechanical properties for the intended applications.

To further investigate the preservation of mechanical properties, additional tests were conducted to evaluate the changes in Young’s modulus following the immersion and exposure of the samples to low temperatures. Based on the results obtained previously, the two-day immersion period was selected, followed by exposure to low temperatures (−80 °C) for an additional two days. The tensil tests in Figure 2 show a Young’s modulus of 6.03 ± 0.13 MPa, corresponding to a reduction of 25.02% compared to the initial reference value.

### 3.3. Analysis of 3D-Printed Surface Modifying Printing Orientation

To test the effects of print orientation on surface morphology, 3D-printed samples were designed with simplified geometries and printed using three different orientations relative to the print bed (0°, 45°, and 90°).

The 3D-printed samples were characterized using a Mitaka profiler to analyze the surface roughness by varying the printing angle. These measurements also allowed to obtain accurate references to detect roughness values after water immersion and exposure to low temperatures, which is described in the following paragraphs.

**Figure 3:**
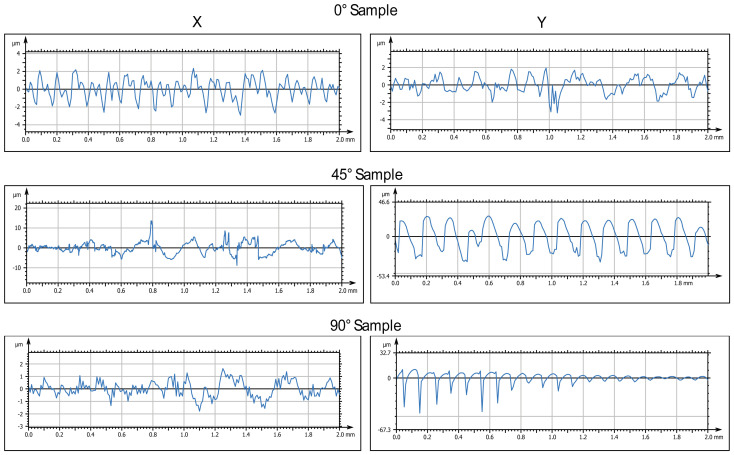
Two-dimensional surface analysis of printed samples before immersion: representative images of roughness profiles (Ra, µm), taken by a Mitaka noncontact surface profiler, for samples printed at three different orientations (0°, 45°, and 90° with respect to the printing plane).

**Figure 4:**
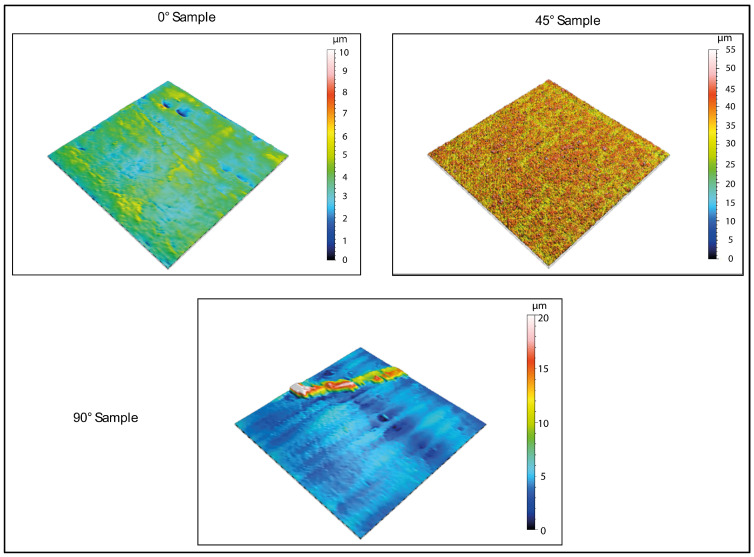
Three-dimensional surface analysis of printed samples before immersion (reference values). The analyses carried out show that the surfaces, before immersion and treatment at low temperatures, are free of defects, except for those derived from the printing process.

Figure 3 and Figure 4 display the 2D and 3D surface scans of the samples, respectively. Results indicate that samples printed at 45° and 0° angles showed higher surface roughness, correlating with the sample growth direction. For these angles, layer alignment along the printing axis caused discontinuities, increasing surface irregularities. Consequently, morphology and surface roughness varied according to printing orientation. This is evident in the 2D representations, where surface layers are visible, particularly along the sample growth direction (Figure 3, y axis). These layers are not detectable in the X-axis scan, as the printing process is aligned with the Y-axis. In contrast, the sample printed at 90° exhibited more homogeneous surface roughness in both scanning directions, indicating a uniform distribution of surface imperfections and reduced growth direction dependence. These findings suggest that print orientation influences the surface properties of elastomeric 3D-printed samples, impacting mechanical performance and environmental interactions.

### 3.4. Analysis of 3D-Printed Surface After Immersion and Exposure at Low Temperature

**Figure 5:**
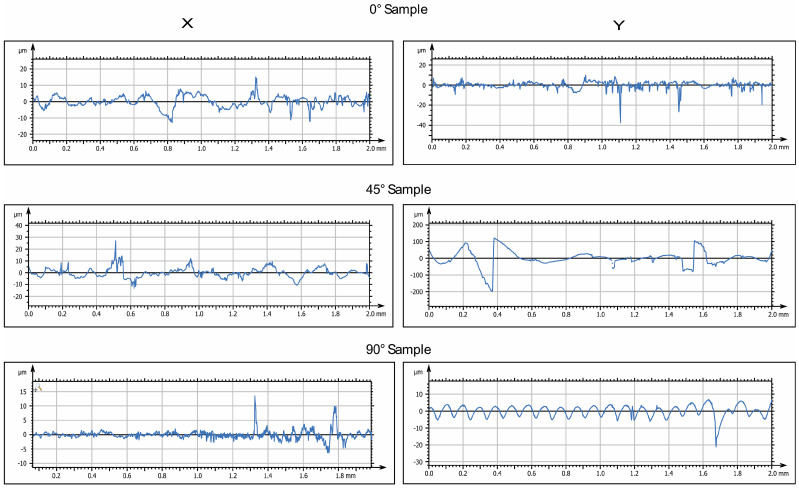
Two-dimensional surface analysis of samples after thermal exposure at −20 °C. Representative images of roughness profiles (Ra, µm), taken by a Mitaka noncontact surface profiler, for samples printed at three different orientations (0°, 45° and 90°, with respect to the printing plane).

**Figure 6:**
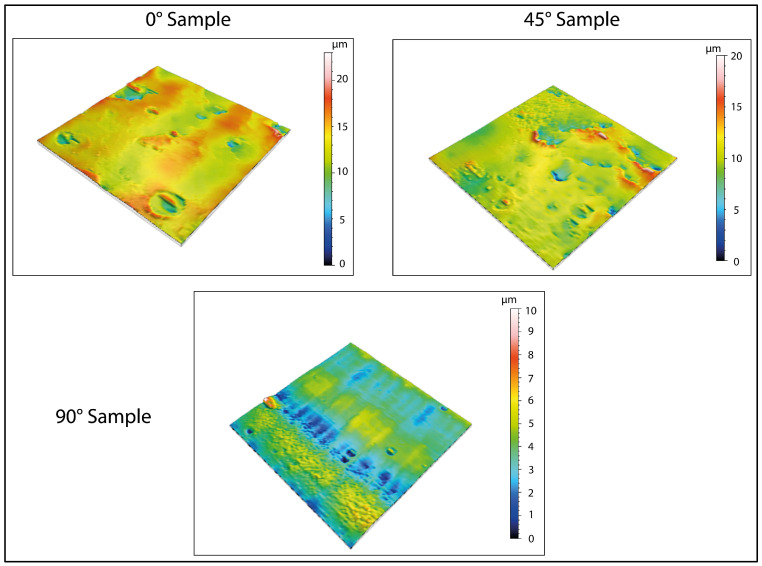
Three-dimensional surface analysis of samples after thermal exposure at −20 °C. The acquisition shows that, after the first treatment at low temperatures (−20 °C), pore formation occurs on the surface, caused by the expansion of water during the transition to a solid state.

At the end of the immersion phase for two days in water, the samples were taken, shaken of excess water, and transferred in a freezer at −20 °C for two days. To evaluate the variation in the surface aspect, analysis was performed by a profiler. The evolution of surface characterizations is shown in Figure 5 and Figure 6 (in 2D and 3D, respectively).

From the results obtained, an increase in the surface roughness was observed for all the orientations considered. However, this increase was more pronounced for the sample printed at 0°. The increase in roughness was confirmed by 3D maps (Figure 6). It is important to note that this increase was not uniform but was instead concentrated in specific areas of the specimen, corresponding to porous formation.

**Figure 7:**
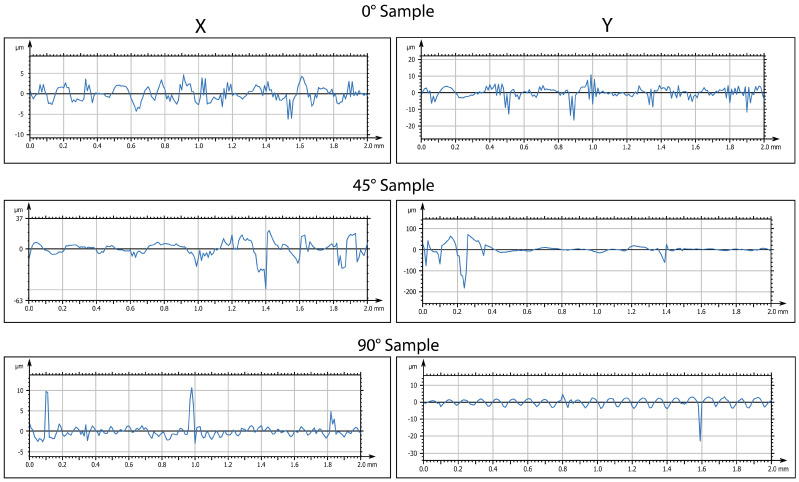
Two-dimensional surface analysis of samples after thermal exposure at −80 °C: typical roughness profiles (Ra, µm). Representative images of roughness profiles (Ra, µm), taken by a Mitaka noncontact surface profiler, for samples printed at three different orientations (0°, 45°, and 90° with respect to the printing plane).

**Figure 8:**
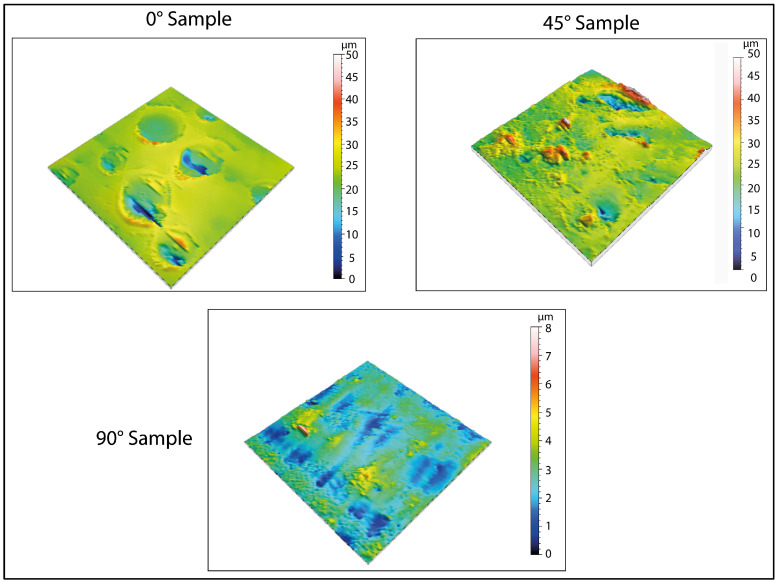
Three-dimensional surface analysis of samples after thermal exposure at −80 °C. The acquisition shows that, after the second treatment at low temperatures (−80 °C), pore formation occurs on the surface, caused by the expansion of water during the transition to the solid state.

A further cooling phase was implemented. In this phase, the samples were maintained at −80 °C for 2 days. Treatment at −80 °C aims to induce an extreme cooling of the microstructure. The authors supposed that such a low temperature would facilitate the formation of larger pores, particularly in the most susceptible areas of the specimen. After the completion of this phase, the samples underwent surface analysis to determine morphological evolution. Defects of higher sizes are visible in Figure 7 and Figure 8.

### 3.5. Variation in Average Roughness as a Function of Print Orientation and Temperature

The evolution of surface morphologies is shown in Table 8 and Table 9 in terms of linear roughness (Ra) and surface roughness (Sa).

**Figure 9:**
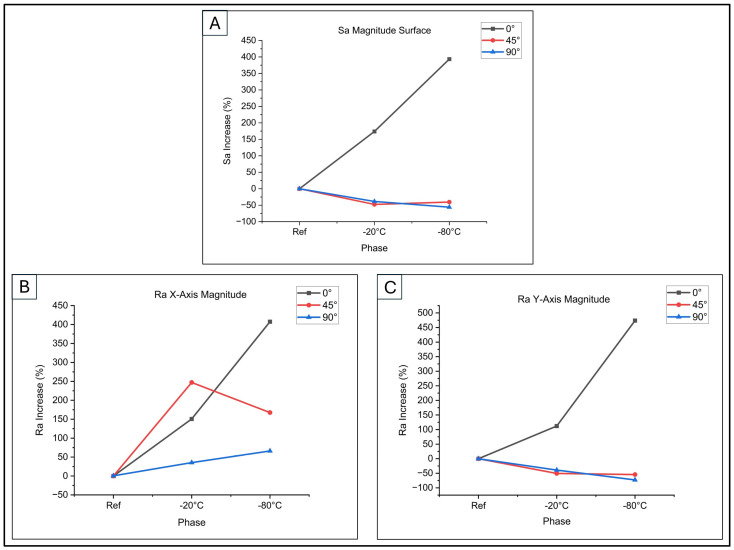
Surface analysis: (**A**) surface roughness (Sa%); (**B**) Ra% along X-axis; (**C**) Ra% along Y-axis.

By applying Equations (3) and (4), the percentage variations in both linear and surface roughness were obtained. They are graphed in Figure 9.

(3) $${Ra}_{\%}=\left(\frac{{Ra}_{ref}-{Ra}_{-20{}^{\circ}C,-80{}^{\circ}C}}{{Ra}_{ref}}\right)\times 100$$

(4) $${Sa}_{\%}=\left(\frac{{Sa}_{ref}-{Sa}_{-20{}^{\circ}C,-80{}^{\circ}C}}{{Sa}_{ref}}\right)\times 100$$

Figure 9A illustrates these trends for surface roughness during low-thermal exposure. Surface roughness (Sa%) increased in both the first phase (−20 °C) and the second phase (−80 °C) for 0° samples. Conversely, 45° and 90° samples showed a reduction in surface roughness for both phases. This analysis highlights the impact of printing orientation and thermal exposure on surface topography, crucial for cell adhesion and proliferation.

As depicted in Figure 9B (building direction along the X-axis), samples at 0° and 45° orientations demonstrated a marked rise in linear roughness Ra% following low-temperature treatment. The increase was particularly notable, reaching 150.83% and 247.16% after exposure at −20 °C, and 407.5% and 167.61% following treatment at −80 °C. This phenomenon can be explained by the condensation and rigidification of polymer chains at reduced temperatures, resulting in heightened surface irregularity, according to [37]. Conversely, the 90° sample exhibited less pronounced roughness increases, with values of 35.42% initially (−20 °C) and 66.15% subsequently (−80 °C).

Examining Figure 9C, which depicts the building direction along the Y-axis, it can be observed that only specimens printed at 0° exhibit an increase in linear roughness Ra%. This increase was initially 112.04%, followed by a further rise of 473.77% after exposure to low temperatures. Conversely, samples printed at 45° and 90° demonstrated a reduction in Ra%. Following treatment at −20 °C, these samples showed decreases of 50.44% and 38.86% respectively, while treatment at −80 °C resulted in reductions of 54.36% and 72.85%. These variations in surface roughness may be attributed to changes in the surface structure, which are influenced by the diverse material orientations used during the printing process and subsequent interactions with low temperatures.

**Figure 10:**
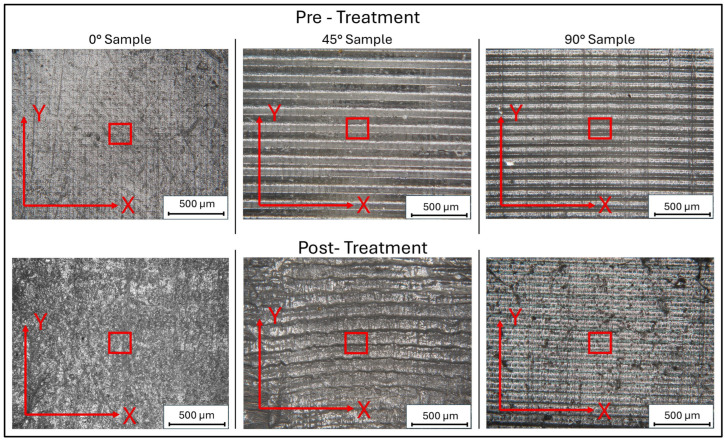
Representative images of samples before and after treatment at low temperatures@ magnification of 160×. Data in the red areas were acquired by 3D scans.

Comparative analysis of the sample surfaces before and after treatment, utilizing 160× magnification with the Hirox microscope, revealed macroscopic variations, particularly for 0° samples (Figure 10). We observed that the 0° sample exhibited plastic deformation in the central area. In this region, a marked increase in surface roughness was observed compared to the other areas, which appeared to be more homogeneous. This phenomenon can be attributed to the presence of surface defects or printing imperfections, which facilitate the accumulation of water, thus resulting in the plastic deformation of the surface.

Moreover, from both surface maps and images at 160× magnification, it is possible to discern the formation of pores of different sizes, which could improve the functionality of the scaffold in tissue engineering applications.

The variation in surface roughness observed in the 45° and 90° samples, compared to the 0° samples, can be attributed to the interaction of multiple factors. Specifically, this variation is associated with the presence of surface defects, which may be pre-existing or form during sample preparation, and with the residual stress generated by gradients of temperature during the low-temperature treatment. Immersion in water can induce differential swelling on the material’s surface, while cooling may cause contractions that generate residual stress [37]. The combination of these effects increases roughness, which is more pronounced in the 0° samples, as shown in Figure 9, while it is more contained in 45° and 90° samples.

Consequently, the effect of thermal stress emerges as a determining factor in the variation in surface roughness, with a significantly reduced impact on samples with orientations not parallel to the printing surface (0° samples).

Regarding the formation of pores, the 3D maps presented in Figure 6 and Figure 8 reveal the emergence of surface porosity when compared to the reference maps in Figure 4. These pores, however, lack uniformity in both geometry and surface distribution. The findings suggest that the printing angle significantly affects pore distribution. This phenomenon can be attributed to localized variations in the material’s microstructure, resulting in the uneven absorption of the immersion liquid by the surface. Such heterogeneity influenced the expansion of the liquid during low-temperature treatment, leading to the development of pores with irregular shapes.

Table 10 shows the values relating to the average pore diameters, subject to standard deviation (SD), as a function of the three printing inclinations and the two temperatures analyzed.

For specimens printed at a 0° angle, the pores show a more uniform distribution across the surface, indicating a more consistent formation process. Additionally, the pores have a relatively regular shape, even though there are significant variations in their average size. This uniformity can be attributed to the simpler geometric structure of the surface associated with this printing orientation, as demonstrated by the three-dimensional image in Figure 4. Conversely, samples printed at a 45° angle demonstrate irregular pore morphologies and non-uniform distributions. This phenomenon may be caused by various factors, including residual stress generated during printing and the inherent complexity of the surface morphology in this configuration. These conditions could have influenced the absorption of the immersion liquid, subsequently affecting its expansion during low-temperature treatment.

For samples printed at a 90° angle, pore formation is restricted and only occurs after exposure at −80 °C. This phenomenon can be attributed to the smaller surface area that is exposed to the effects of ice expansion, which limits pore creation.

## 4. Conclusions

Scaffolds play a vital role in biomedical research and tissue engineering, as they provide a three-dimensional environment that mimics in vivo conditions, allowing the creation of in vitro models that more accurately simulate the native environment than traditional two-dimensional cell cultures [38]. Recognizing the importance of elasticity in many tissue types, this study focused on developing a method to improve elastomeric scaffold performance by tailoring surface porosity.

Preliminary analyses were conducted to investigate the behaviour of the elastomeric resin, focusing on swelling and changes in Young’s modulus. Results showed that swelling stabilized after three days for both immersion liquids, with higher values seen in distilled water than in saline solution. Mechanical tests showed a decrease in Young’s modulus during the first days of immersion, with a subsequent convergence after five days. Additionally, tests conducted on samples exposed to low temperatures showed minimal changes in Young’s modulus, ensuring the preservation of the mechanical properties of the material.

The protocol was applied by immersing the 3D-printed samples (with different orientations) in distilled water, followed by exposure to low temperatures (−20 °C and −80 °C). This process induced porosity formation and increased surface roughness, with the pore extent and distribution influenced by both printing orientation and temperature. Samples printed at 0° showed pores with an average diameter of 17.03 ± 2.56 µm and more uniform porosity, especially after exposure to −80 °C, suggesting that this combination could be optimal for scaffold fabrication. Indeed, the presence of micropores in scaffolds supports cell adhesion, cell–cell and cell–matrix signalling, promoting the self-organization of cells into functional tissue structures [22,35,39]. Further studies on cell adhesion and proliferation will be addressed in future works.

In conclusion, this research highlights the importance of optimizing elastomeric scaffold design by considering printing orientation, material properties, and environmental parameters such as temperature.

## Figures and Tables

**Table 1:** Monomers in SLA Resin.

Polymer/Monomer	Percentage of Composition (%)
3,3,5-TrimethylCyclohexyl Methacrylate	10–20
Methacrylate Ester Monomer	70–90
Dimetacrilate di Uretano	<5
Phenylbis (2,4,6-TrimethylBenzoyl) Phosphineoxide (BAPO)	<2

**Table 2:** Parameters for the tensile test.

Parameter	Value
Velocity (mm/min)	500 (constant)
Thickness (mm)	3.00
Length (mm)	50.8
Acquisition Frequency (Hz)	1.00
Max Load (N)	100

**Table 3:** Printing parameters for sample production.

Parameter	Value		
Layer thickness (mm)	0.05		
Exposure temp (s)	45.00		
Exposure off (s)	4.00		
Exposure first layer (s)	120.00		
Number of first layers	5		
Type of adhesion	Raft		
Type of support	Tree		
Density of support (%)	0° Sample	45° Sample	90° Sample
	2.50	3.00	1.00

**Table 4:** Parameters used for 2D surface scanning.

Parameter	Value
Direction of analysis	X and Y axis
Pitch (µm)	1.00
Range (mm)	2.00
Scanning speed (µm/s)	10.00
Autofocus	On (Standard)
Objective lens	LM 100X

**Table 5:** Parameters for 3D surface scanning.

Parameter	Value
Pitch asse X (µm)	1
Pitch asse Y (µm)	1
X-axis length (μm)	100
Y-axis length (μm)	100
Scanning speed (µm/s)	10.00
Autofocus	On (standard)

**Table 6:** Swelling progression as a function of immersion time in water and physiological solution (NaCl 0.9). The values are represented as an average ± SD, n = 6.

Time	2 Days	3 Days	5 Days	7 Days
Water (%)	3.36 ± 0.71	6.74 ± 0.22	7.37 ± 0.60	7.73 ± 0.10
NaCl 0.9 (%)	1.59 ± 0.04	1.79 ± 0.34	2.32 ± 0.23	2.90 ± 0.15

**Table 7:** The Average Young’s modulus obtained for each dive time and percent change from the non-dived reference sample (0 days). The values are represented as an average ± SD of three sample for every dive time.

	0 Days	2 Days	5 Days	7 Days
Young Module (MPa)	8.73 ± 0.85	6.37 ± 1.02	4.06 ± 0.30	4.33 ± 0.62
*E*_%_	0	27.02	53.43	50.31

**Table 8:** Linear roughness (Ra) as determined by digital roughness analyses. The values are represented as an average ± SD of six acquisitions for each sample.

	Ref Ra (µm)		−20 °C Ra (µm)		−80 °C Ra (µm)	
	X	Y	X	Y	X	Y
0°	0.90 ± 0.08	0.81 ± 0.11	2.26 ± 0.60	1.72 ± 0.52	4.57 ± 0.33	4.65 ± 0.21
45°	0.88 ± 0.08	16.4 ± 0.15	3.06 ± 0.48	8.13 ± 0.87	2.36 ± 0.94	7.49 ± 0.98
90°	0.48 ± 0.08	5.81 ± 0.10	0.65 ± 0.17	3.55 ± 0.79	0.80 ± 0.11	1.58 ± 0.13

**Table 9:** Surface roughness (Sa) as determined by digital roughness analyses.

	Ref Sa (µm)	−20 °C Sa (µm)	−80 °C Sa (µm)
0°	0.46	1.26	2.27
45°	2.08	1.09	1.24
90°	1.22	0.75	0.54

**Table 10:** Average pore diameters obtained using 3D-printed surfaces at tilt angles of 0°, 45° and 90°. The values are represented as an average ± SD of ten acquisitions for each sample.

Exposure Temp	−20 °C	−80 °C
Parameters	Average Diameter (µm)	Average Diameter (µm)
0°	12.06 ± 4.59	17.03 ± 2.56
45°	6.93 ± 2.12	10.20 ± 3.14
90°	5.67 ± 2.57	15.93 ± 4.30

## Data Availability

The original contributions presented in this study are included in the article. Further inquiries can be directed to the corresponding authors.
